# Eyelid and brow asymmetry in patients evaluated for upper lid blepharoplasty

**DOI:** 10.1186/s40463-014-0036-4

**Published:** 2014-10-02

**Authors:** Kristian I Macdonald, Adrian I Mendez, Robert D Hart, S Mark Taylor

**Affiliations:** Department of Otolaryngology – Head & Neck Surgery, University of Ottawa, 1081 Carling Ave, Ottawa, ON K1Y 4G2 Canada; Division of Otolaryngology – Head & Neck Surgery, University of Alberta, Edmonton, AB Canada; Division of Otolaryngology – Head & Neck Surgery, Dalhousie University, Halifax, NS Canada

**Keywords:** Eyelid, Eyebrow, Symmetry, Asymmetry, Blepharoplasty, Informed consent, Patient evaluation

## Abstract

**Introduction:**

In evaluation for blepharoplasty, patients often desire improved cosmesis and/or correction of visual field deficits. However, patients are usually unaware of eyelid or brow asymmetry. Furthermore, the prevalence of eyelid and brow asymmetry is infrequently reported in the medical literature.

**Purpose:**

To determine the prevalence of brow and eyelid asymmetry in patients evaluated for upper lid blepharoplasty.

**Methods:**

One hundred consecutive patients evaluated for upper lid blepharoplasty were included in the study. Standard pre-operative photographs were taken of all patients using consistent background and photographic equipment. Two of the authors (KM & AM) independently recorded the margin pupil (MPD), central eyebrow (CED), nasal eyebrow (NED) and temporal eyebrow (TED) distances. To test the inter-observer reliability, the senior author (SMT) recorded the same measurements for 10% of randomly selected patients. We calculated 95% confidence intervals to compare symmetry between the right and left sides.

**Results:**

One hundred patients (94 female, mean age 57.7) were included in the study. The average MPD, CED, NED and TED distances were 0.55 mm (95% CI 0.45-0.65), 1.77 mm (95% CI 1.47-2.07), 1.34 mm (95% CI 1.14-1.54), and 1.78 mm (95% CI 1.50-2.06), respectively. Ninety-three percent of patients had at least one asymmetric measurement of greater than 1 mm. Seventy-five percent of patients studied had at least one measurement greater than 2 mm while 37 percent had at least one greater than 3 mm.

**Conclusion:**

Brow and eyelid asymmetry is common in patients being evaluated for upper lid blepharoplasty. The facial plastic surgeon should identify and document facial asymmetry pre-operatively, and discuss it with prospective blepharoplasty patients. This will improve informed consent and patient expectations.

## Background

In evaluation for blepharoplasty, patients often desire improved cosmesis and/or correction of visual field deficits. However, patients are usually unaware of eyelid or brow asymmetry [1-3]. This is interesting to note, given the importance of facial symmetry in defining beauty [4,5].

Preoperatively, it is important to identify and inform the patient of the presence of eyelid and brow asymmetry [1,6]. This will allow for a comprehensive surgical plan, and will help ensure reasonable patient expectations. Although there is no clear consensus on what degree of asymmetry is of clinical importance, some authors report that facial asymmetry as little as 1 mm is of significance and warrants attention [7-9].

The prevalence of eyelid and brow asymmetry is infrequently reported in the medical literature. Song et al. reported a 30% asymmetry of the palpebral fissure in a random population of 594 Koreans [8]. A study examining photos of models in popular magazines found that 10% had asymmetry of several measures of eyelid and brow height [9].

It is the senior author’s (SMT) hypothesis that a significant proportion of patients presenting for evaluation for blepharoplasty have eyelid and/or brow asymmetry. This group is particularly interesting to study, as although they are inquiring about eyelid surgery, most are unaware of any existing asymmetry.

## Methods

This retrospective chart review was approved by the local research ethics board at Dalhousie University in Halifax, NS, Canada. We included consecutive patients who were evaluated for upper lid blepharoplasty. Patients were excluded if they had a documented eyelid or brow asymmetry with a defined cause, including previous surgery and/or facial nerve palsy.

**Figure 1:**
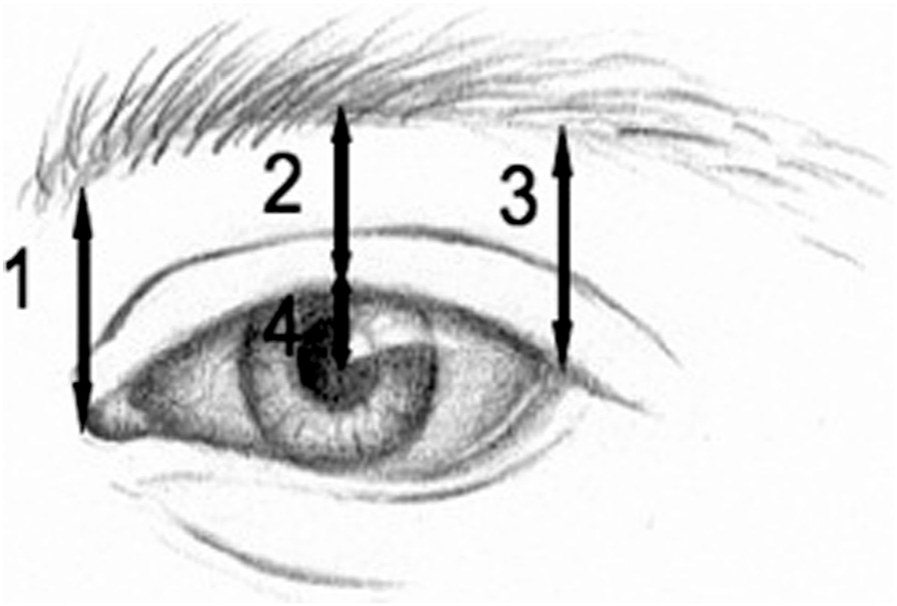
**Definitions of the measurements used in this study. 1**. Nasal eyebrow (NEP), the distance from the medical canthus to lower eyebrow. **2**. Central eyebrow (CEP), the distance from the upper lid margin to the lower eyebrow in the mid-pupil plan. **3**. Temporal eyebrow (TEP), the distance from the lateral canthus to the lower eyebrow. **4**. Margin pupil distance (MPD), the distance from the central upper lid margin to the centre of the pupil.

Preoperative pictures were taken in a standardized fashion with the same camera, at zero magnification, at the same distance from the patient. From these pictures, a senior (KM) and a junior Otolaryngology – Head & Neck Surgery resident (AM) independently measured the four distances detailed in Figure 1 for each eye. These were the margin pupil (MPD), the central eyebrow (CED), the nasal eyebrow (NED), and the temporal eyebrow (TEP) distances.

For each measurement, the mean asymmetry with 95% confidence interval was calculated. We also determined the proportion of patients who had equal to or greater than 1, 2 and 3 mm asymmetry for each measurement. To test for inter-observer reliability, 10% of the patients were randomly selected for independent measurements by the senior author, a facial plastic surgeon (SMT). These measurements and asymmetries were compared to those of the other two authors.

## Results

One hundred consecutive patients who presented for evaluation for upper lid blepharoplasty were included in the analysis. None of the patients were excluded. There were 97 Caucasians and 3 Asian patients in the study cohort. 94 patients were female, and 6 were male. The average age of the group was 57.7 +/- 10 years.

The 10 sets of randomly selected patients measured by the senior author were compared to those of the first author. Although some of the actual measurements varied slightly, the asymmetries were within 0.1 mm for all 40 measurements.

The proportions of patients with ≥1, 2 and 3 mm of asymmetry are presented in Table 1. In summary, 93% of patients had greater than or equal to 1 mm of asymmetry in at least one of four measurements, 75% had greater than or equal to 2 mm, and 37% had greater than or equal to 3 mm.

**Table 1:** **Number of patients with asymmetry with 3 different limits**

**Measurement**	**≥1 mm**	**≥2 mm**	**≥3 mm**
**MPD**	11	3	1
**CED**	58	38	18
**NED**	50	25	10
**TED**	61	41	18
**≥1 measurement of asymmetry**	93	75	37

MPD = Margin pupil distance; CED = Central eye distance; NED = Nasal eye distance; TED = Temporal eye distance.

**Figure 2:**
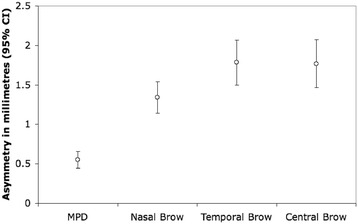
**Average eyelid and brow asymmetries for 100 patients.** MPD = Margin pupil distance.

Figure 2 reports the average asymmetry, in millimeters, for each of the four measurements. The average MPD, NED, TED and CED were 0.55 mm (95% CI 0.44-0.66), 1.34 mm (95% CI 1.14-1.54), 1.78 mm (95% CI 1.49-2.07), and 1.77 mm (95% CI 1.46-2.07), respectively.

**Figure 3:**
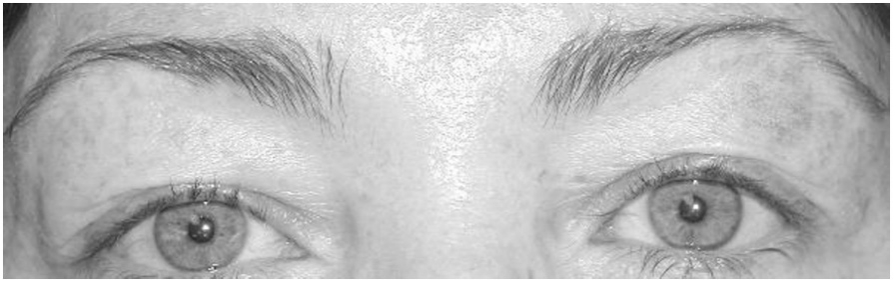
**Patient 1.** Asymmetries: MPD = 0 mm; CED = 1.5 mm; NED = 1.5 mm; TED = 4 mm.

**Figure 4:**
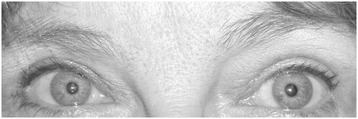
**Patient 2.** Asymmetries: MPD = 0 mm; CED = 5.5 mm; NED = 6 mm; TED = 3.5 mm.

Two examples are presented in Figures 3 and 4. Both patients were females in their early 40′s who had requested a more youthful appearance of their upper eyelids. The first patient (Figure 3) had a 4 mm asymmetry in the temporal eye distance, with the right side higher than the left. The second patient (Figure 4) had a more obvious asymmetry, with a central eye distance asymmetry of 5.5 mm. Neither patient noted their asymmetry as a complaint or reason for requesting surgery.

## Discussion

Published literature on facial analysis has stressed the importance of preoperatively identifying facial asymmetry [1-3,10]. However, the actual prevalence of eyelid and brow asymmetry is rarely reported. Searches online in Medline with the terms “eyelid asymmetry”, “brow asymmetry”, “photo analysis and eyelids”, and “facial asymmetry and aesthetics”, yielded few relevant studies.

Ing et al. evaluated 102 models in popular magazine photographs [9]. They measured 14 ocular parameters, and identified a mean asymmetry of 0.2-2.4 mm. 12 of the models had an asymmetry of 2 standard deviations from the mean, concluding that a significant number of models had substantial facial asymmetry.

In another study, Song et al. sought to quantify asymmetry of palpebral fissure height in normal Koreans [8]. They recruited 594 patients from the general population, and determined the prevalence of asymmetry greater than 1 mm. They found that 24.2% of males and 26.5% of females had such an asymmetry. The authors called for similar studies in Caucasians.

Our patient population is particularly interesting, as they are mostly female Caucasians (94%) who sought surgical correction of the upper eyelid. Although they had paid particular attention to their eyes, they were not aware of, or at least did not make note of any asymmetry during their clinic visit. We could not identify other studies in which eyelid or brow asymmetry was assessed in patients presenting for blepharoplasty.

Despite this, the vast majority of our patients (93%) had, in at least one of the four measurements, asymmetry greater than or equal to 1 mm. Using more stringent criteria, three quarters of patients had an asymmetry greater than or equal to 2 mm, in at least one of the measurements.

Awareness of the prevalence of asymmetry is important for the facial plastic surgeon. It will enhance the preoperative evaluation, help optimize the surgical plan and improve patient expectations and satisfaction. Once their other concerns are addressed, patients who were not aware of asymmetries preoperatively may be more likely to take notice postoperatively, and could conclude that they are iatrogenic in etiology. It is therefore critical that the facial plastic surgeon identifies potential asymmetry preoperatively and educates the patient appropriately.

## Conclusion

The vast majority of patients presenting for evaluation for upper lid blepharoplasty had eyelid or brow asymmetry greater than or equal to 1 mm. Temporal and central eyebrow distances showed the greatest asymmetry. This knowledge is critical for the facial plastic surgeon, and any potential asymmetry should be identified and discussed preoperatively with blepharoplasty patients.

### Consent

Written informed consent was obtained from the patient for the publication of this report and any accompanying images.
